# Internal validation protocol for large collaborative clinical data sets: assessment of the CONGRESS database

**DOI:** 10.1308/rcsann.2025.0094

**Published:** 2026-01-20

**Authors:** K Cole, JA Gossage, P Bhandari, NS Blencowe, S Chidambaram, T Crosby, RPT Evans, EA Griffiths, SK Kamarajah, SR Markar, N Trudgill, TJ Underwood, PH Pucher

**Affiliations:** ^1^ Portsmouth Hospitals University NHS Trust, UK; ^2^ Guy’s and St Thomas’ Hospital NHS Foundation Trust, UK; ^3^ University of Bristol, UK; ^4^ Imperial College London, UK; ^5^ Velindre University NHS Trust, UK; ^6^ University Hospitals Birmingham NHS Foundation Trust, UK; ^7^ University of Birmingham, UK.; ^8^ University of Oxford, UK; ^9^ University of Southampton, UK; ^10^ University of Portsmouth, UK

**Keywords:** Oesophagogastric cancer, Early oesophageal cancer, Collaborative research, Data validation

## Abstract

**Introduction:**

Multicentre clinical research collaboratives collect large, generalisable data sets. However, data are often collected by trainees who may lack clinical or academic experience, raising concerns about data quality and potential reporting bias. Validation practices in such studies are variable. This study outlines the methods, feasibility, and outcomes of internal data validation using the CONGRESS database.

**Methods:**

The multicentre CONGRESS data set of early oesophagogastric cancer was assessed. A random 20% sample of patients was selected to meet a >15% target validation size. Patient, disease and outcome data were re-abstracted from medical records and entered into a validation data set, which was compared with the original database. Cohen’s kappa coefficient (κ) and Pearsons corelation (*r*) were calculated to express the strength of agreement between categorical and continuous variables, respectively.

**Results:**

In total, 302 patients (18.1%) from the original CONGRESS database were included in the validation data set and 3,320 data points were compared between data sets (6,640 total). The percentage of exact agreement for variables ranged from 82.5% to 98.7% (median 92.3%, interquartile range 86.3%–95.7%). Nine variables (1,645 of 2,946, 55.8% data points) showed ‘almost perfect’ agreement (κ or *r* > 0.8), and five (1,301 of 2,946, 44.2%) showed substantial agreement (κ > 0.6). None showed weak or poor agreement.

**Conclusion:**

This study proposes a reproducible framework and benchmarks for validating large collaborative clinical data sets, using the national CONGRESS data set as an example. This approach offers a standard for ensuring reliable, high-quality research outcomes across multicentre databases.

## Introduction

Multicentre clinical research collaboratives are used to generate large clinical data sets and high-impact research.^1–3^ This promotes the rapid and efficient generation of multicentre and often multinational data, producing more diverse and representative data sets.^4^ Compared with single-site studies, such data are more reflective of broader populations, enhancing the generalisability and clinical relevance of research findings.^5^ Collaborative data sets are especially valuable for investigating rarer conditions, where large sample sizes are necessary to achieve sufficient statistical power.^6^

These collaboratives, however, commonly rely upon a multitude of individuals, including trainees and medical students, to collect data. These individuals typically receive minimal training and may lack the clinical expertise required for accurate data interpretation. Furthermore, data entry is frequently performed with limited oversight, raising concerns about the accuracy and consistency of the resulting data sets. Without formal validation, the reliability of these databases remains uncertain and may compromise the credibility of subsequent research findings.

The current practice for validating these collaborative databases is variable and lacks standardisation. Several collaborative research groups, such as GlobalSurg and STARSurg, have successfully validated prospective studies.^7–13^ In doing so they reported centre ascertainment rates of 48%–100%, and overall data accuracy of up to 99.2%. National registries such as the Dutch Upper Gastrointestinal Cancer Audit, the Swedish National Register for Oesophageal and Gastric Cancer (NREV) and the Swedish Colorectal Cancer Register have also reported high internal validity and reliable data.^14–16^ However, the techniques used, and the extent of validation and quality control, vary widely.

This study addresses the need for greater consistency in validating collaborative data sets. The CONGRESS database, a UK-led, multicentre, multinational resource on early oesophagogastric (OG) cancer, was used to assess data accuracy and reproducibility through a structured validation framework.^6^ By describing this methodology in detail, the study highlights a reproducible approach that could support the standardisation of validation practices in future collaborative research.

## Methods

The CONGRESS (endosCopic resectiON, esophaGectomy or gastrectomy foR Early oeSophagogastric cancerS) database is a multicentre retrospective cohort of patients undergoing curative treatment for T1N0 OG adenocarcinoma diagnosed between 2015 and 2022. Collected data included patient demographics, clinical diagnosis, management and pathology. The full inclusion and exclusion criteria have been previously described.^6,17^ Data were identified, screened and inputted by trainees under the nominal supervision of named lead surgeons for each centre; ultimately data for 1,673 patients across 28 centres in total were included.

Internal validation of CONGRESS data was undertaken by adapting established methods previously applied to national registry data sets.^14–16,18,19^ A target sample size of 15% of the original database was set prior to data analysis, based on registry validation studies.^14–16,18,19^ To account for centres dropping out or being unable to participate, the initial request to participants randomly selected 20% of each centre’s original data through a computerised random number generation process. Only centres that included >30 patients in the original data set were approached, because these centres would otherwise be asked to return only five or fewer patients in the validation set with a risk of poor data completeness at centre level. Anonymised patient, disease, treatment and outcome data were then collected from the original medical records and resubmitted into an online database (Research Electronic Data Capture [REDCap]) identical to the original data set. In the validation series, data collectors were different individuals from those involved in the original data collection, and were blinded from the original data. All validators were specialist surgical trainees and were provided with clear written guidelines for data collection and one-on-one technical and clinical advice. The study was locally reviewed by each appropriate institutional authority and registered as a retrospective service evaluation of anonymised outcome data.

### Statistical analysis

Clinically relevant integer and categorical variables were compared between the validation and original data sets. These included patient demographics, tumour variables, treatment outcomes, and follow-up data. The chosen variables were set prior to data analysis.

Internal validity was initially expressed as the percentage of exact agreement in both data sets. Cohen’s kappa coefficient (κ) was then used to measure the strength of the agreement for categorical variables (weighted κ for ordinal variables and unweighted κ for nominal variables), and Pearsons correlation coefficient (*r*) was used for continuous variables. Qualitative interpretation for both was as per Landis and Koch and Evans, whereby <0.20 indicated ‘slight agreement’, 0.21–0.40 ‘fair agreement’, 0.41–0.60 ‘moderate agreement’, 0.61–0.80 ‘substantial agreement’ and 0.81–1.00 ‘almost perfect agreement’.^20,21^

Allowances were made for temporal differences between survival status at the time of the original and validation data sets, because the validation data set was collected 18 months after the original data set; a data disagreement was recorded if a patient was initially recorded as dead in the initial data set, but alive in the validation data set. Given anecdotal problems with the correct calculation and inputting of patient age at diagnosis, we also allowed a ±1 year tolerance on patient age when assessing exact agreement. All other datapoints were compared with absolute values.

To assess whether sample size had any effect on data accuracy, the correlation between *r* for age and centre sample size was assessed. All data analyses were performed using the SPSS v30 (IBM Corp, Armonk, NY, USA).

## Results

Data for a total of 302 patients from 13 centres, and 3,320 data points were included for validation, representing 18.1% of the original CONGRESS database. The percentage of exact agreement across the 17 variables was high, ranging from 82.5% to 98.7% (median 92.3%, interquartile range 86.3%–95.7%), with low rates of missing data (Table 1). Of the 13 categorical variables tested, 7 showed ‘almost perfect’ and 5 showed ‘substantial’ agreement.

**Table 1:** Missing data, exact agreement and correlation for validated variables (in comparison with the CONGRESS database)

Variable		Missing data CONGRESS	Missing data VALIDATION	*n* (excluding missing or non-reported data)	Agreement of non-missing data (%)
Demographics	Age^*^	0/302	0/302	302	84.4
	Gender	0/302	0/302	302	95.4
	Charlson score 0	0/302	0/302	302	82.5
Clinical staging		8/302	4/302	290	83.4
Index treatment		5/302	5/302	296	96.3
Type of ER		0/250	0/259	249	95.2
ER pathology	T stage	1/250	1/259	248	87.5
	LVI status	16/240	0/243	211	95.7
	Differentiation	20/240	3/243	190	86.3
	R1 resection (deep)	0/240	1/243	221	92.3
Surgical pathology	T stage	1/85	1/76	71	88.7
	LVI status	2/85	0/76	69	95.7
	Differentiation	3/60	2/54	50	84
	Nodal status	0/85	0/76	73	97.3
	Total node harvest	0/85	1/76	72	91.7
	R1 resection (circumferential)	0/85	1/76	72	98.7
Status at last follow-up		4/302	2/302	302	94.6

ER = endoscopic resection; LVI = lymphovascular invasion

*Allows for age within validation data to be 12 months higher or lower than original data

**Figure 1:**
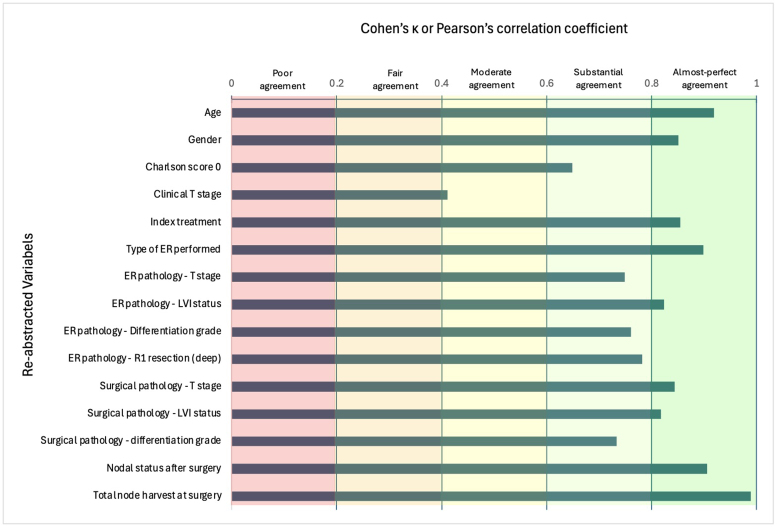
Cohen’s kappa coefficient score for all validated variables. Qualitative interpretation of the kappa coefficients was as per Landis and Koch^20^ : κ = 0–0.20 (slight agreement); 0.21–0.40 (fair agreement); κ = 0.41–0.60 (moderate agreement); κ = 0.61–0.80 (substantial agreement); and κ = 0.81–1.0 (almost perfect agreement). ER = endoscopic resection; LVI = lymphovascular invasion

**Figure 2:**
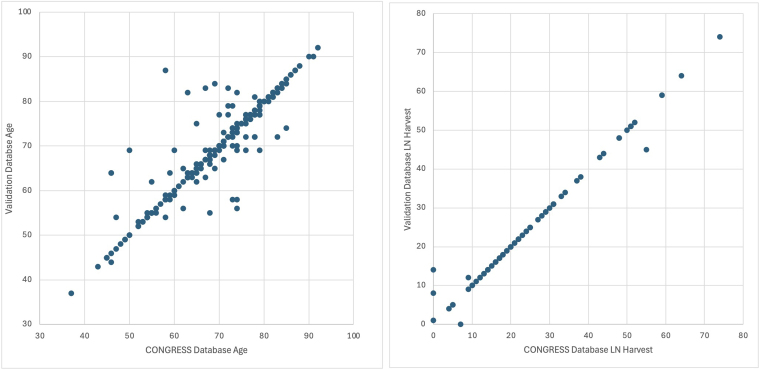
Exact agreement and correlation between validation database and original CONGRESS database for (a) age at diagnosis and (b) lymph node harvest at surgery. LN = lymph node

The highest levels of agreement were seen for index treatment (κ = 0.854, 96.3% absolute agreement), gender (κ = 0.851, 95.4%) and type of endoscopic resection (κ = 0.898, 95.2%) (Figure 1). The lowest level of agreement was for clinical T stage, with moderate agreement (κ = 0.411, 82.5%). Correlation was very strong for the continuous variables age (*r* = 0.919, 84.4%) and nodal harvest (*r* = 0.989, 91.7%) (Figure 2a,b).

The nature of data discrepancies was assessed visually (Tables 2–4) and did not suggest a systematic pattern of error, with a random distribution of data discrepancies (e.g. no over-staging or under-staging bias).

**Table 2:** Exact agreement and correlation between validation database and original CONGRESS database for patient demographics and management

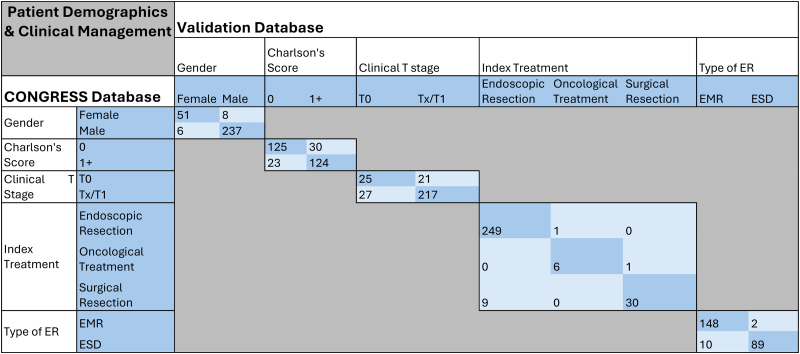

EMR = endoscopic mucosal resection; ER = endoscopic resection; ESD = endoscopic submucosal dissection; LVI = lymphovascular invasion

**Table 3:** Exact agreement and correlation between validation database and original CONGRESS database for endoscopic resection pathology

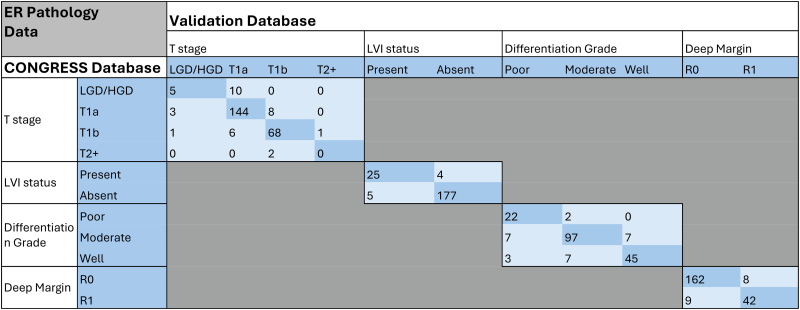

ER = endoscopic resection; LVI = lymphovascular invasion

**Table 4:** Exact agreement and correlation between validation database and original CONGRESS database for surgical resection pathology

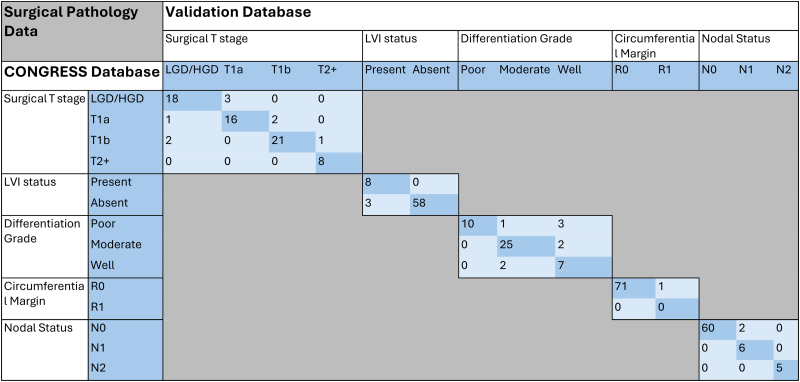

HGD = high-grade dysplasia; LGD = low-grade dysplasia; LVI = lymphovascular invasion

Correlation between centre sample size and age correlation coefficient was poor (*r* = −0.067, *p* = 0.828), suggesting no association (bias) between data accuracy and centre sample size (see Appendix 1, available online, for plot of correlation coefficient *r* vs centre sample size).

## Discussion

This study has successfully demonstrated the high validity and reproducibility of the CONGRESS database, reinforcing the credibility of both its previously published and future findings. These results align with those of previous validations of clinical data sets.^7–13^ The structure and governance of the CONGRESS data set shared similar approaches and steering group personnel with other UK-based studies such as the Oesophago-Gastric Anastomotic Audit.^2^ The methodology and evidence presented here serve to strengthen the validity of these and other similarly structured collaborative projects in general.

In current literature, the methods for validating collaborative databases lack standardisation. Similar to previous Swedish registry studies, we assessed discrepancies at the level of individual variables, allowing for the detection of systematic over- or under-reporting of continuous or ordinal data; for example, TNM staging. The lack of directional bias observed suggests consistent data entry (or error) over time, despite potential changes in data collectors, clinical systems or documentation practices. By contrast, some collaborative research groups choose not to express their validation data at the individual variable level. Instead, they report overall accuracy by calculating the proportion of correct data points across all validated entries within a set selection of variables.^9,13^ Although this approach provides a valuable and broad overview of data set accuracy, it might be argued that examining individual, clinically relevant variables is important to ensure that specific data elements do not unduly influence study results. In addition, there is inconsistency in the proportion of cases being validated, with some studies selecting as little as 5%–10% of data.^8,9,12^

Other published validation approaches include having the lead investigators at each site manually review their centre’s data prior to submission.^11^ However, this method may introduce bias and is often impractical in larger data sets, where a single person cannot feasibly verify every entry. The GlobalSurg group adopted a more rigorous approach, combining pre-data collection training with retrospective assessments of case ascertainment and data accuracy.^7,10^ In one study, this group assessed case ascertainment in 66 of the 343 participating hospitals and employed independent validators to evaluate the data accuracy. Kappa values ranged from 0.651 to 0.934. However, validation was hindered by high levels of missing data, particularly in low-income settings, with some variables showing up to 42.2% unavailable data.^10^ Such figures suggest that although overall clinical research collaboratives can result in very good data accuracy, the interhospital variability warrants validity assessment for data quality assurance and transparency.

### Study strengths and limitations

This study has demonstrated a robust technique for maintaining and verifying high-quality data in a large, retrospective collaborative database, summary recommendations for which can be found in Table 5. A key strength of this study lies in its robust validation methodology. De novo data were collected independently by a new group of individuals, with information extracted directly from original medical records. This approach minimised potential bias and demonstrated the reproducibility of data across independent collection cycles.

**Table 5:** Recommendations for maintaining and verifying data quality in research collaboratives

Recommendations for clinical research collaborative data validation
Primary data collection
Designated senior data collection lead at each centre Predefined input variables and formatting (ideally into centralised online database) Secured storage of local pseudonymisation tables for audit
Data validation
Validation across all contributing centres Randomised selection of 15% of all patients/cases Validation data collector independent from original study collector Replicate primary data collection form in validation data set Threshold kappa for categorical and Pearson correlation for continuous data ≥ 0.7 Where thresholds not met, reevaluate at centre or variable level for a correctable process or issue, else consider excluding data

A learning point and potential limitation was that two centres were unable to submit validation data because of missing local pseudonymisation keys. Although both centres and local principal investigators were highly experienced collaborative researchers, this highlights the importance of identifying and confirming local data repositories. Although only higher volume centres were included in the validation study (to avoid inclusion of centres with five or fewer patients, a number which would typically merit exclusion from statistical analysis), the absence of a significant association between agreement and sample size suggests this is unlikely to have relevance to the overall findings. A further limitation is that case ascertainment was not evaluated; this is typically assessed by comparing submitted data volumes against administrative data sets. However, this presumes accuracy of administrative coding, and is in any case not possible in the assessment of disease (sub)types that are not uniquely coded for, such as early OG cancer. Future research could investigate factors that might influence data accuracy, including the experience or training of data collectors, or other local resources.

This study supports the validity of a now commonly used approach in large research collaboratives: predefined variables, anonymised data entry into structured electronic databases with mandatory fields, and local maintenance of a pseudonymisation key for audit and governance purposes. However, larger multicentre studies, or those involving complex data sets designed to address multiple research questions, should warrant individual validity assessment to ensure data quality and reliability. For future validity assessments, we would propose a threshold kappa or Pearson coefficient of >0.7, or data agreement level of >80%–90%. Below this level, detailed investigation and correction of the source of data discrepancy should be considered. If related to an individual centre or contributor, these may require training and data assessment, or exclusion. If related to a specific variable, the study protocol or database inclusion criteria may require clarification to standardise interpretation and entry. If a specific pattern of error can be identified (e.g. over- or under-staging), it may relate to temporal changes in the database or patient condition. In the absence of a correctable issue, the validity of the database or collaborative personnel as a whole may need be called into question.

## Conclusions

In summary, this study introduces a practical and reproducible framework for validating collaborative clinical databases, with proposed benchmarks and recommended actions. Although these data sets are increasingly being used for high-impact research, without robust validation they face inherent challenges and can be questioned on their data consistency and accuracy. Using the CONGRESS database, we have demonstrated a validation approach that enhances confidence in collaborative research outputs and supports their continued development.

## Author contributions

**K Cole**: Data curation, Formal analysis, Investigation, Methodology, Writing – original draft, Writing – review & editing. **JA Gossage**: Conceptualisation, Data curation, Writing – review & editing. **P Bhandari**: Conceptualisation, Data curation, Writing – review & editing. **NS Blencowe**: Conceptualisation, Data curation, Writing – review & editing. **S Chidambaram**: Conceptualisation, Data curation, Writing – review & editing. **T Crosby**: Conceptualisation, Data curation, Writing – review & editing. **RPT Evans**: Conceptualisation, Data curation, Writing – review & editing. **EA Griffiths**: Conceptualisation, Data curation, Writing – review & editing. **SK Kamarajah**: Conceptualisation, Data curation, Writing – review & editing. **SR Markar**: Conceptualisation, Data curation, Writing – review & editing. **N Trudgill**: Conceptualisation, Data curation, Writing – review & editing. **TJ Underwood**: Conceptualisation, Data curation, Writing – review & editing. **PH Pucher**: Conceptualisation, Data curation, Formal analysis, Investigation, Methodology, Project administration, Supervision, Validation, Writing – original draft, Writing – review & editing. All authors have read and approved the final manuscript.

## CONGRESS collaborative co-authors:

Tarig Abdelrahman, Khalid Akbari, Hiba Al-Bahrani, Leo Alexandre, Hasan Ali, Bilal Alkhaffaf, Anuradaha Alwis, Antonios Athanasiou, Evan Best, Khalid Bhatti, Nick Bird, Matthew Boal, Alex Boddy, Matt Bonomaully, Amir Botros, George Bouras, Leo Brown, Benjamin Byrne, Richard Byrom, Beatriz Carrasco Aguilera, David Chan, Clarisa TP Choh, Martina Hermez Chole, Hollie Clements, Peter Coe, Andrea Cross, Fiona Crotty, Vinutha Dayashetty, Niall Dempster, Alexander Dermanis, Massimiliano Di Pietro, Simon Dwerryhouse, Ahmed Elshaer, Nada Elzahed, Hisham Elzanati, Sarah Epton, Matthew Forshaw, Lewis Gall, Ismael Ghazzi, Leeying Giet, Hasan Haboubi, George B Hanna, Paul Healy, Jonathan Hoare, Sung Hong, Faisal Ibrahim, Anchal Jain, Chenchen Ji, Courtney Johnson, Victoria Karibo-Alalibo, Sharib Khan, Fredrik Klevebro, Mie Thu Ko, Bhaskar Kumar, Ponette Abigail Lau, Jie Lim, Steven Lindley, Anantha Madhavan, Ashuvini Mahendran, Henrik Maltzman, Michel Martin, Sotiris Mastoridis, Leo McCormick Matthews, Euan McLaughlin, David Mitton, Krishna Moorthy, Gael Nana, Magnus Nilsson, J Robert O'Neill, Mervyn Owusu-Ayim, Sally Pan, Simon Parsons, Pradeep Patil, Ian Penman, Abeerah Pervez, Christopher Peters, Shaun Preston, Oliver Priest, Saqib Rahman, Sarveson Rajkumar, Tom Ritchie, Ioannis Sarantitis, Azita Shahdoost-Rad, Negar Sharafi, Katie Siggens, Aayush Sinha, Richard Skipworth, Naim Slim, Maria Soupashi, Sophie Stevens, Jennifer Straatman, Vikas Sud, Jav Sultan, Cheuk-Bong Tang, Nav Thavanesan, Paul Turner, Bhamini Vadhwana, Ravi Vohra, Shajahan Wahed, Michael White, Thomas Whittaker, Vincent Wong, Susannah Woodrow.

## Supplementary Information

The online version contains supplementary material available at https://doi.org/10.1308/rcsann.2025.0094.
